# Unsafe Care and Fake News in Freeman–Burian Syndrome

**DOI:** 10.1002/ccr3.70078

**Published:** 2025-01-06

**Authors:** Mikaela I. Poling, Craig R. Dufresne

**Affiliations:** ^1^ Craig R Dufresne Fairfax Virginia USA

**Keywords:** case reports, craniocarpotarsal dystrophy, craniofacial abnormalities, distal arthrogryposis type 2A, Freeman–Burian syndrome, Freeman–Sheldon syndrome, malignant hyperthermia, rare diseases, whistling face syndrome

## Abstract

Freeman‐Burian syndrome is a rare craniofacial syndrome surrounded by fake news. This situation shows the strong connection between the quality of a literature search and clinical reasoning displayed in patient care, especially in care of patients with rare conditions.

We read with great interest the article by Sato (Boku), Sento, Hasegawa, Tsutsumi, Kamimura, So, Kako, and Sobue, “Anesthetic management of a patient with Freeman‐Sheldon syndrome undergoing oral surgery: a case report” [1, 2]. It is encouraging to see this exquisitely rare condition discussed in the literature. While undoubtedly well‐intentioned and methodical, there are a number of concerns (Table 1).

**TABLE 1:** Major errors in case report of Freeman–Burian syndrome published in June 2021 and rationale.

Authors' error	Rationale
Reference list	Lack of recent references, including: articles on the genetic cause, clinical diagnosis, a meta‐analysis, anesthesia recommendations
“reported prevalence than 1 per 1 million”	Based on one flawed study and no longer accepted. Poor writing
“multiple joint contractures, characteristic facial features, such as microtia, defects of the hands and feet, such as clubfoot, and skeletal malformations”	Joint contractures and extremity deformities are nonspecific findings. Only the four craniofacial findings of microstomia, pursed whistling lips, deep nasolabial folds, and H or V shaped chin defect are pathognomonic for FBS. FBS is a myopathy, not skeletal condition. Microtia is not seen in FBS
Omission of the clinical diagnostic criteria	Not stating the diagnostic criteria confuses the reader unfamiliar with FBS
“association with malignant hyperthermia (MH),” and FBS	MH is not associated with FBS
“Reports on the general anesthetic management of FSS patients have been disorganized…”	Fails to mention meta‐analysis or clinical practice recommendations
Omission of photographs or a description of how the patient met the diagnostic criteria	Stating the patient had FBS is insufficient, considering the false positive rate may be between 30%–60%
General anesthesia would cause, “worsening of respiratory insufficiency and postoperative pneumonia.”	Good anesthesia care avoids both
The authors considered four options: local anesthesia, local anesthesia with narcotic analgesia, local anesthesia with sedation, and general endotracheal anesthesia	The two safe options are local anesthesia only and general endotracheal anesthesia. Sedation without a secure airway should not happen, as dysphagia, pulmonary complications (especially aspiration pneumonia), and difficult airway are all major problems in this patient population. While respiratory depression can be exacerbated with opiates, short‐acting opiates have been safely used.
The authors considered three concerns involving general endotracheal anesthesia: difficult intubation, “respiratory failure,” and risk of MH	Difficult intubation is a major challenge in FBS but not a contraindication. “Respiratory failure” should never be a likely event with good care. Respiratory depression is a greater concern in FBS but can be prevented. MH is not associated with FBS
“causes respiratory decline in adults”	No evidence of decline or (if present) of FBS as a primary cause.
“respiratory muscle fatigue”	Primary muscle fatigue is not part of FBS.
“There was no facial deformity or limitation of retroflexion in this case.”	Craniofacial deformities are required for FBS diagnosis.
“Although NHF was not used in this case, it may have been useful…”	Anatomic infeasibility due to narrowed nasopharynx

Most areas of concern seemed to be related to an insufficient search of the recent literature on Freeman–Sheldon syndrome, now Freeman–Burian syndrome (FBS) [3]. For example, the authors omitted seminal articles that defined the clinical and genetic diagnosis, [4, 5] molecular physiology [6, 7, 8], the only meta‐analysis on FBS [9], and a clinical recommendation series that includes evaluation, anesthesia care, and dental and orofacial treatment [10, 11, 12]. Unfortunately, the authors cite [1, 2] only one recent article, which was one of 12 flawed case reports published since 2020 [13] to which we responded [14]. As FBS is an exquisitely rare condition, little is known about it. Many who believe they have encountered it in clinical practice are eager to publish their experience. Special care is required in the selection of articles from the literature on FBS, as much of it contains oft repeated fallacies.

The unsubstantiated FBS frequency of 1:1 million, derived from a retrospective database study of UK patients with skeletal dysplasias, was cited [1, 2, 15]. FBS is often misdiagnosed, making it inadvisable to accept a recorded FBS diagnosis without objective patient data [9]. This study's frequency for FBS is no longer accepted, and the prevailing estimate is that 200–300 individuals worldwide may have FBS [16].

Sato (Boku) et al. describe patients with FBS as having, “multiple joint contractures, characteristic facial features, such as microtia, defects of the hands and feet, such as clubfoot, and skeletal malformations” [1, 2]. External ear position variances may be seen in FBS, but microtia is not seen in FBS [9]. Otherwise, the description is mostly true for most patients, while remaining misleading. Only the craniofacial features (microstomia, pursed whistling lips, deep nasolabial folds, and H or V shaped chin defect) of the diagnostic criteria are pathognomonic for FBS [4, 5, 9]. Distal extremity contractures are nondiagnostic findings in FBS and common in many syndromic and nonsyndromic entities [4, 9, 16]. The authors also do not state [1, 2] the accepted clinical diagnostic criteria [4] that have been shown to be strongly correlated with molecular diagnosis [5]. Not directly stating the diagnostic criteria can confuse the reader unfamiliar with FBS.

FBS is a congenital craniofacial syndrome of myopathic origin that frequently involves findings outside the craniofacial region (spine and extremities), though FBS has had many classifications since its first description in 1938 and independent confirmation in 1962 [17, 18, 19]. Contrary to Sato (Boku) et al. “skeletal malformations” are secondary effects of the primary myopathic process of fibrose tissue replacement of normal muscle fibers [16]. This fibrose tissue acts as constricting bands, the way collagen behaves in severe burns [16]. These findings are consistent with in vitro molecular myophysiology observations showing problems with the metabolic process for contraction and extreme muscle stiffness that reduces muscular work and power [5, 6, 7]. Misunderstanding of etiology in FBS has led to inappropriate treatment plans, especially surgeries, and has resulted in tragic, lifelong impairments [9, 16, 20].

The authors referred to a risk of, “association with malignant hyperthermia (MH),” and FBS [1, 2]. The potential association of MH and FBS was based on a single report of two cases [21]. Some patients with FBS do, indeed, develop hyperpyrexia during general anesthesia, but it has also been observed to be resolved by administration of ibuprofen [11]. These hyperpyrexia events, which may include tachycardia and increased muscle rigidity, have also been seen in settings where an MH protocol was followed and in non‐operative situations of physical or mental stress beyond the individual's baseline [11]. It is, now, believed these hyperpyrexia events are not MH events and that MH is not associated with FBS or the DAs [11].

The authors write that, “Reports on the general anesthetic management of FSS patients have been disorganized” [1, 2]. While it is true that anesthesia case reports have been spotty and of varying quality, this is not a unique phenomenon [9]. There are only two published studies of FBS, neither of which addressed anesthesia in any detail [4, 5]. There is, however, a meta‐analysis of individual patient data extracted from rigorously evaluated case reports and anesthesia clinical practice recommendations [9, 11]. Both of these articles consolidate the evidence‐base for anesthesia care of FBS patients in detail [9, 11].

Without photographs or a detailed description of how the patient met the diagnostic criteria, it is not certain the patient described had FBS. Stating the patient had FBS is insufficient, considering the false positive diagnosis rate may be 30%–60% [9]. Near the end of the article, the authors remark that, “There was no facial deformity… in this case,” [1, 2], making it unlikely the patient descried had FBS. Four main craniofacial deformities (microstomia, pursed whistling lips, deep nasolabial folds, and H or V shaped chin defect) are required for diagnosis [4, 5, 9], and all patients present with an assortment of additional common but not required craniofacial stigmata [9, 10].

The authors assert that general anesthesia would cause, “worsening of respiratory insufficiency and postoperative pneumonia” [1, 2]. Though the risk is greater for postoperative respiratory complications in FBS patients, properly managed general anesthesia certainly does not directly cause postoperative respiratory distress and pneumonia [11]. Nonetheless, patients require care from experienced providers, ideally in tertiary referral centers [11]. Astute anesthesia and postanesthesia care for patients with FBS, as outlined in the clinical practice recommendations, is specifically directed toward avoiding postoperative respiratory sequelae [11].

In their management decision‐making, the authors consider four options: local anesthesia, local anesthesia with narcotic analgesia, local anesthesia with sedation, and general anesthesia [1, 2]. Expressly because of the need to ensure a secure airway to prevent aspiration pneumonia, the two safe options are local anesthesia only, where the patient is fully alert without impaired cognition and able to protect their own airway; and general anesthesia, where the patient's airway is secured via orotracheal intubation or a surgical airway [11, 12]. The authors acknowledge the pulmonary concerns [1, 2, 22] but paradoxically wish to avoid invasive airway management in a sedated patient undergoing oral surgery, but sedation or use of a potentially cognitive‐impairing agent without a secure airway during a procedure presents a major risk to patient safety in FBS and should not happen [1, 2]. Local anesthesia with narcotic analgesia would not be an option either. As correctly observed by Sato (Boku) et al. respiratory depression can be exacerbated with opiates, but short‐acting options have been used safely in this syndrome [11]. In our view, the bigger risk is the cognitive effect of the narcotics when used in an awake FBS patient for procedural pain control for oral surgery [11, 12]. Impaired cognition could inhibit a patient's ability to conscientiously protect their away.

Sato (Boku) et al. outline their three concerns with general anesthesia: difficult intubation, “respiratory failure,” and risk of MH—seemingly as justification for considering general anesthesia contraindicated in FBS or for their case, at least [1, 2]. Difficult intubation is a major challenge in FBS requiring considerable skill but is accomplished under the proper conditions when needed [11]. The other two concerns are flatly illogical. “Respiratory failure” should never be a likely event with good care [11]. While respiratory depression is a greater concern in FBS than for the general patient population, preventing it is a major goal for all anesthesia care [11]. As mentioned above, MH is not associated with FBS [11].

Sato (Boku) et al. write, “[FBS] causes respiratory decline in adults” [1, 2]. There is no evidence supporting either a decline in respiratory function or of FBS being a primary cause in any decline observed. While FBS may limit healthy physical activity necessary for maintaining respiratory function status, lifestyle and aging are expected to be main contributors to any observed decline, as they would be in the general population [20]. The authors also refer to, “respiratory muscle fatigue” [1, 2]. As discussed previously, FBS results in the formation of white fibrous tissue constricting bands within normal muscle and complete muscle replacement by white fibrous tissue [16]. Primary muscle fatigue is not understood to be part of FBS.

Finally, the authors consider nasal high‐flow (NHF) oxygen cannulae in FBS and write, “Although NHF was not used in this case, it may have been useful….” [1, 2]. Though NHF has an important role in critical care and other settings, its use in this patient population probably would be impractical, due to anatomically restricted airflow in the nasopharynx of most FBS patients precluding efficient oxygenation. For this reason, nasal airways and nasal intubation also are ineffective airway management methods for many FBS patients. Orotracheal intubation or a surgical airway are the most reliable techniques for providing effective airway protection and positive‐pressure support [11].

FBS is a rare complex craniofacial syndrome that has much fake news written about it. As illustrated in the present article, the literature search is not a skill restricted to the preserve of academicians. Indeed, there can be a strong connection between the quality of a literature search and clinical reasoning displayed in patient care, especially in the care of patients with rare conditions.

## Author Contributions

**Mikaela I. Poling:** conceptualization, writing – original draft, writing – review and editing. **Craig R. Dufresne:** supervision, writing – review and editing.

## Conflicts of Interest

The authors declare no conflicts of interest.

## Data Availability

The authors have nothing to report.
